# Galgravin Isolated from *Piper kadsura* Ameliorates Lipopolysaccharide (LPS)-Induced Endotoxemia in Mice

**DOI:** 10.3390/ijms242316572

**Published:** 2023-11-21

**Authors:** Shih-Ming Ou, Yin-Chieh Hsu, Shu-Ling Fu, Lie-Chwen Lin, Chao-Hsiung Lin

**Affiliations:** 1 Department of Life Sciences and Institute of Genome Sciences, National Yang Ming Chiao Tung University, Taipei 11221, Taiwan; 2 Department of Surgery, Tri-Service General Hospital, National Defense Medical Center, Taipei 11467, Taiwan; 3 Institute of Traditional Medicine, National Yang Ming Chiao Tung University, Taipei 11221, Taiwan; ansonhsu0100@hotmail.com (Y.-C.H.); slfu@nycu.edu.tw (S.-L.F.); 4 National Research Institute of Chinese Medicine, Ministry of Health and Welfare, Taipei 11221, Taiwan

**Keywords:** *Piper kadsura*, galgravin, inflammation, sepsis, lipopolysaccharide, NF-κB

## Abstract

Sepsis results from uncontrolled inflammation, characterized by cytokine storm and immunoparalysis. To assess whether galgravin, a natural lignan isolated from *Piper kadsura*, can be used to treat sepsis, models of bacterial lipopolysaccharide (LPS)-activated macrophages and LPS-induced endotoxemia mice were used. Galgravin suppressed NF-κB activation in LPS-activated RAW 264.7 macrophages without causing significant cytotoxicity, in which proinflammatory molecules like TNF-α, IL-6, iNOS, and COX-2 were downregulated. In addition, the expression of TNF-α and IL-6 was also suppressed by galgravin in LPS-activated murine bone marrow-derived macrophages. Moreover, galgravin significantly downregulated the mRNA expression of TNF-α, IL-6, and iNOS in the lungs and decreased TNF-α and IL-6 in the serum and IL-6 in the bronchoalveolar lavage fluid of LPS-challenged mice. The COX-2 expression in tissues, including the lung, liver, and kidney, as well as the lung alveolar hemorrhage, was also reduced by galgravin. The present study reveals the anti-inflammatory effects of galgravin in mouse models and implies its potential application in inflammation diseases.

## 1. Introduction

Sepsis is a host immune disorder manifested with systemic inflammatory response syndrome (SIRS), whereas severe sepsis leads to septic shock and multiple organ dysfunction syndrome (MODS). The three-month mortality rate among patients with septic shock is nearly 50% [1]. Therefore, a significant effort is made to prevent the progress of sepsis to septic shock, which is the overture of death. Upon sepsis-induced MODS, the lung, liver, and kidney are vital organs that are severely affected. In the lung, the pathogenesis of sepsis-induced injury includes damage to the vascular endothelium and alveolar epithelium [2]. The pathogenesis of sepsis-induced injury in the liver involves the dysfunction of sinusoidal endothelium by bacterial toxins and hypoperfusion [3]. The underlying mechanism of sepsis-induced injury in the kidney comprises damage to the renal endothelium and renal tubular epithelium [4].

The biological role of inflammation is eradicating invading pathogens and promoting tissue repair. However, dysregulated inflammation and cytokine storms may occur in severe sepsis. Lipopolysaccharide (LPS), an endotoxin derived from the outer membrane of Gram-negative bacteria, is often used as a pathogen-associated molecular pattern (PAMP) to induce inflammation. Toll-like receptor 4 (TLR4) is a receptor for LPS [5] and is mainly expressed in myeloid immune cells, including macrophages, but also in some non-immune cells like adipocytes and endothelial cells [6]. The following two major pathways are involved in LPS-induced TLR4 downstream signaling: the MyD88-dependent and TRIF-dependent signaling pathways. The former is initiated by TLR4 activation in the plasma membrane, whereas the latter is activated after TLR4 internalization into the endosomes.

In the MyD88-dependent signaling pathway, LPS binds to the TLR4/MD-2 complex and induces TLR4 activation, whereas TIRAP located at the cytoplasmic side of the TLR4/MD-2 complex further recruits MyD88, interleukin-1 receptor-associated kinases (IRAK) 1 and IRAK2 to form a submembrane complex called the myddosome. The assembled myddosome subsequently recruits an E3 ubiquitin ligase, TRAF6, which promotes TAK1 to phosphorylate and activate IκB kinases α/β (IKKα/β), leading to the subsequent NF-κB activation and translocation to the nucleus to function as a transcription activator. Simultaneously, another downstream signaling molecule of TRAF6/TAK1, mitogen-activated protein kinase (MAPK), is also activated and promotes the activities of transcription factors AP-1 and CREB. As a result, the MyD88-dependent signaling pathway positively regulates the downstream genes encoding tumor necrosis factor α (TNF-α), interleukin-6 (IL-6), nitric oxide synthase (iNOS), and cyclooxygenase-2 (COX-2) [5].

Alternatively, the endosome results from the endocytosis of TLR4 and contains an adaptor molecule TRIF and a ubiquitin ligase TRAF3 on the surface. This complex recruits interferon regulatory factor 3 (IRF3), which is subsequently phosphorylated by TBK1, dissociated from TRIF and translocated to the nucleus. As a result, the TRIF-dependent signaling pathway positively regulates the downstream genes encoding type I interferons, CCL5/RANTES, and CXCL10/IP-10 [5].

**Figure 1:**
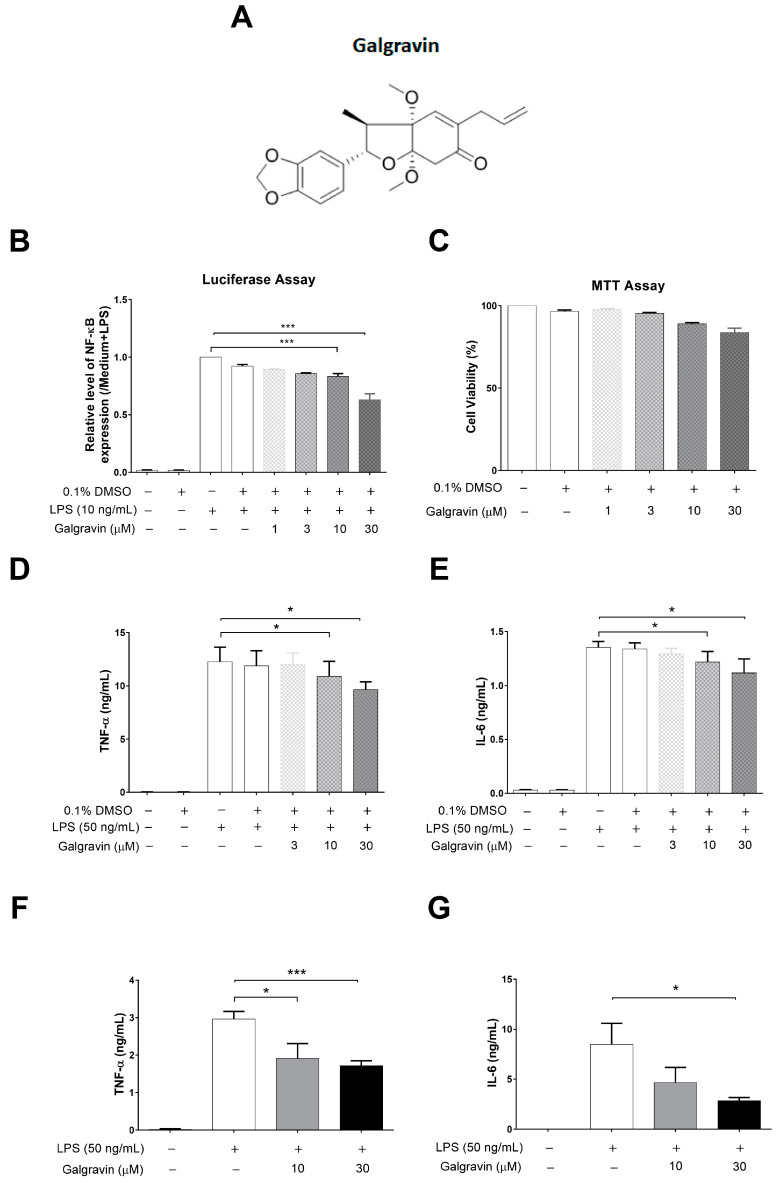
Galgravin inhibits the LPS-induced NF-κB activation and attenuates the production of proinflammatory cytokines in LPS-treated RAW 264.7 cells and BMDM. (**A**) The chemical structure of galgravin. (**B**) RAW 264.7/Luc-P1 cells (4 × 10^5^ cells/well in MP-24) were treated with a vehicle (0.1% DMSO) or different concentrations of galgravin for 1 h, followed by LPS (10 ng/mL) stimulation for 6 h. The resulting cells were lysed in a passive lysis buffer, and the cell lysates were analyzed for luciferase activity. (**C**) RAW 264.7 cells (1 × 10^4^ cells/well in MP-96) were treated with different concentrations of galgravin for 24 h, and MTT assays were performed to determine cell viability. (**D**,**E**) RAW 264.7 cells (3 × 10^5^ cells/well in MP-24) were treated with a vehicle (0.1% DMSO) or different concentrations of galgravin for 1 h, followed by LPS (50 ng/mL) stimulation. Expression levels of TNF-α and IL-6 were measured using ELISA at 3 h and 8 h after LPS treatment, respectively. (**F**,**G**) BMDM cells (1 × 10^6^ cells/well in MP-6) were treated with a vehicle (0.1% DMSO) or different concentrations of galgravin for 1 h, followed by LPS (50 ng/mL) stimulation. The expression levels of TNF-α and IL-6 were measured by ELISA at 3 h and 8 h after LPS treatment, respectively. All the quantification data were from three independent experiments and are shown as the mean ± SEM. The asterisks indicate significant differences from the LPS-treated vehicle control (*, *p* < 0.05; ***, *p* < 0.001). BMDM, bone marrow-derived macrophages. TNF-α, tumor necrosis factor receptor-α. IL-6, interleukin-6.

Many natural products with anti-inflammatory activity have been identified from medicinal plants, such as andrographolide [7,8], curcumin [9], fisetin [10], gossypol [11], and lycorine [12], suggesting that herbal medicine has therapeutic potential for treating sepsis [13]. *Piper kadsura* is a vine-like plant in southeast China and Taiwan [14,15,16], whereas its stem is widely used as a folk medicine for the treatment of rheumatoid arthritis, gouty arthritis, asthma, and cerebral ischemia [14]. Several bioactive ingredients of *P. kadsura* have been identified [14]. Among them, galgravin (Figure 1A) is a tetrahydrofuran lignin [15,17,18,19,20,21,22,23] exhibiting a neuroprotective effect in primary neuronal cultures [24], a cytotoxic effect in human leukemia tumor cells [19], and the inhibitory effect of excessive bone resorption in mouse bone marrow cells [22]. In addition, galgravin also exhibits an edema-suppressive effect in rats with the carrageenan-induced paw test [25], implying its anti-inflammatory potential. However, the mechanism of galgravin against the sepsis model has not yet been reported. In this study, we further investigated the anti-inflammatory activities and molecular mechanisms of galgravin using LPS-activated macrophage cell models and an LPS-induced endotoxemia mouse model.

## 2. Results

### 2.1. Galgravin Inhibits NF-kB Activity in LPS-Treated RAW 264.7 Macrophages

NF-κB is a crucial transcriptional activator of proinflammatory molecules in LPS-activated macrophages, and we further investigated whether galgravin regulates NF-κB activation. A previously established LPS-responsive macrophage cell line, RAW 264.7/Luc-P1, was applied to measure NF-κB activity by assessing luciferase activity [26]. Upon pretreatment with various concentrations of galgravin for 1 h and the following LPS stimulation for 6 h, RAW 264.7/Luc-P1 cells exhibited the galgravin-mediated suppression of NF-κB activity in a concentration-dependent manner (Figure 1B). Moreover, galgravin at effective NF-κB-suppressive concentrations did not cause significant cytotoxicity in RAW 264.7/Luc-P1 cells (Figure 1C). The effect of galgravin on LPS-induced NF-κB activity was measured at 6 h because the effects observed at early time points were less likely to face interference with any potential cytotoxicity caused by more prolonged drug treatment.

### 2.2. Galgravin Attenuates the Production of Proinflammatory Molecules in LPS-Activated RAW 264.7 Macrophages and Bone Marrow-Derived Macrophages (BMDMs)

**Figure 2:**
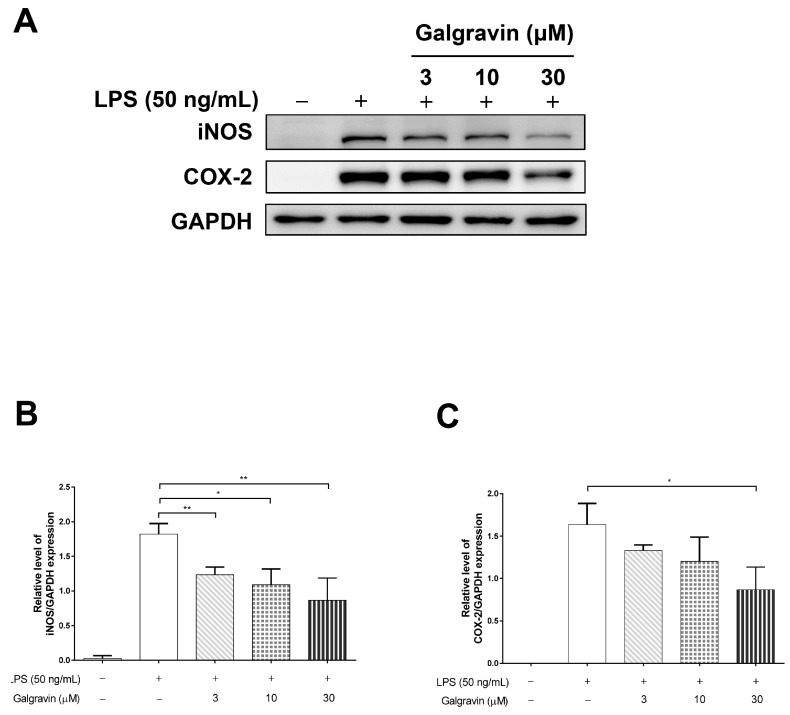
Galgravin attenuates the LPS-induced elevation of iNOS and COX-2 in RAW 264.7 cells. (**A**) RAW 264.7 cells (1 × 10^6^ cells/well in MP-6) were pretreated with a vehicle (0.1% DMSO) or different concentrations of galgravin for 1 h and then incubated with LPS (50 ng/mL) for 24 h. Upon lysis, protein lysates (30 μg) were resolved using 10% SDS-PAGE and subjected to Western blot analysis to determine the expression levels of iNOS and COX-2. (**B**) The quantification data of iNOS from three independent experiments are shown as the mean ± SEM. (**C**) The quantification data of COX-2 from three independent experiments are shown as the mean ± SEM. The asterisk indicates significant differences from the LPS-treated vehicle control (*, *p* < 0.05; **, *p* < 0.01). iNOS, inducible nitric oxide synthase. COX-2, cyclooxygenase 2.

As mentioned above, TNF-α and IL-6 are hallmark proinflammatory cytokines induced by the NF-κB signaling pathway. To determine whether galgravin suppresses LPS-induced inflammation at the molecular level, RAW 264.7 macrophages were pre-treated with increasing concentrations of galgravin (3, 10, and 30 μM) for 1 h, followed by LPS stimulation. As shown in Figure 1C,D, galgravin reduced TNF-α (−1.8% at 3 μM, −11.0% at 10 μM and −21.2% at 30 μM) and IL-6 production (−4.5% at 3 μM, −10.0% at 10 μM and −17.5% at 30 μM) in LPS-stimulated RAW 264.7 macrophages. In addition, galgravin also suppressed TNF-α (−20.1% at 10 μM and −40.9% at 30 μM) and IL-6 (−45.0% at 10 μM and −66.5% at 30 μM) expression in LPS-stimulated BMDMs (Figure 1E,F). The effect of galgravin on the LPS-induced TNF-α and IL-6 expression was measured at 3 and 8 h because the effect measured at early time points was less likely to result from potential cytotoxicity after more prolonged drug treatment. Alternatively, the inducible nitric oxide synthase (iNOS) and COX-2 are two downstream proinflammatory effectors of NF-κB activation. As shown in Figure 2, galgravin suppressed the LPS-induced expression of iNOS (−32.1% at 3 μM, −40.1% at 10 μM and −52.4% at 30 μM) and COX-2 (−18.7% at 3 μM, −26.5% at 10 μM and −47.0% at 30 μM) proteins in RAW 264.7 macrophages. Together, these results indicate that galgravin effectively inhibits LPS-induced proinflammatory cytokines and mediators.

### 2.3. Galgravin Improves the Pathological Phenotypes of LPS-Treated Endotoxemia Mice

**Figure 3:**
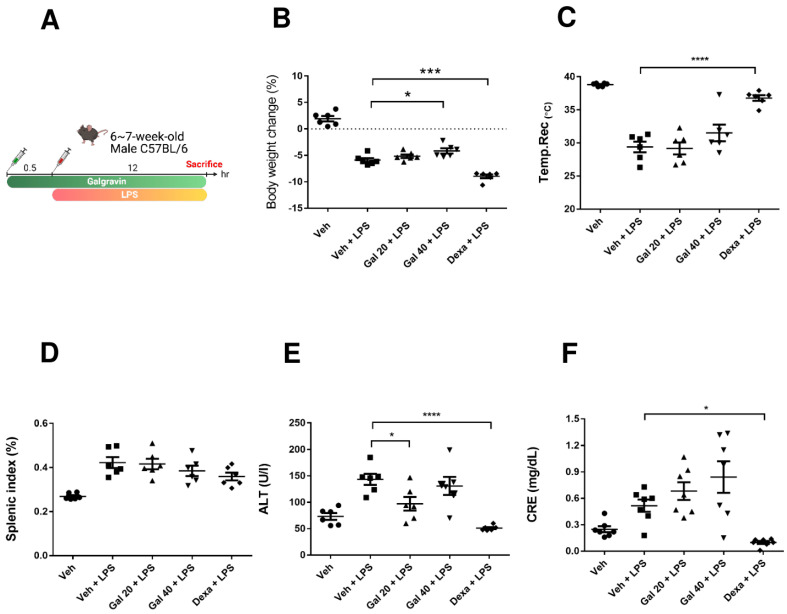
The intraperitoneal administration of galgravin attenuates the production of proinflammatory cytokines in LPS-challenged mice. (**A**) The experimental scheme of the LPS-induced endotoxemia mouse model with galgravin pretreatment. N = 6 for each group. Dexa was used as a positive control. (**B**) Body weight changes in the vehicle-treated mice and LPS-treated mice without or with the pretreatment of drugs were recorded at sacrifice. (**C**) Rectal temperatures of different mouse groups were recorded before sacrifice. (**D**) The splenic index (%) of various mouse groups was calculated from the percentage of the splenic weight to body weight at sacrifice. (**E**) The serum ALT of different mouse groups was measured to evaluate their degree of hepatotoxicity. (**F**) The serum CRE of various mouse groups was measured to assess their degree of nephrotoxicity. Gal 20 and Gal 40 indicate the dosages of galgravin at 20 mg/kg and 40 mg/kg, respectively. The LPS dosage was 20 mg/kg. The dexa dosage was 10 mg/kg. The asterisk indicates significant differences from the LPS-treated vehicle control (*, *p* < 0.05; ***, *p* < 0.001; ****, *p* < 0.0001). Dexa, dexamethasone. ALT, alanine aminotransferase. CRE, creatinine.

We next investigated whether galgravin exerts anti-inflammatory effects in an LPS-induced endotoxemia mice model. Different doses of galgravin (20 or 40 mg/kg) were injected intraperitoneally into C57BL/6 male mice 30 min before the following intraperitoneal injection of LPS (20 mg/kg). After 12 h of the LPS challenge, the mice were sacrificed to collect their lungs, livers, kidneys, spleens, and serum, as depicted in Figure 3A. Our results showed that galgravin (40 mg/kg) treatment reduced body weight loss in LPS-treated mice (Figure 3B). However, the observed improvement of hypothermia, splenomegaly, liver function, and renal function via galgravin did not reach statistical significance (Figure 3C–F).

### 2.4. Galgravin Attenuates the mRNA Levels of IL-6, TNF-α and iNOS in Lung Tissues of LPS-Challenged Mice

**Figure 4:**
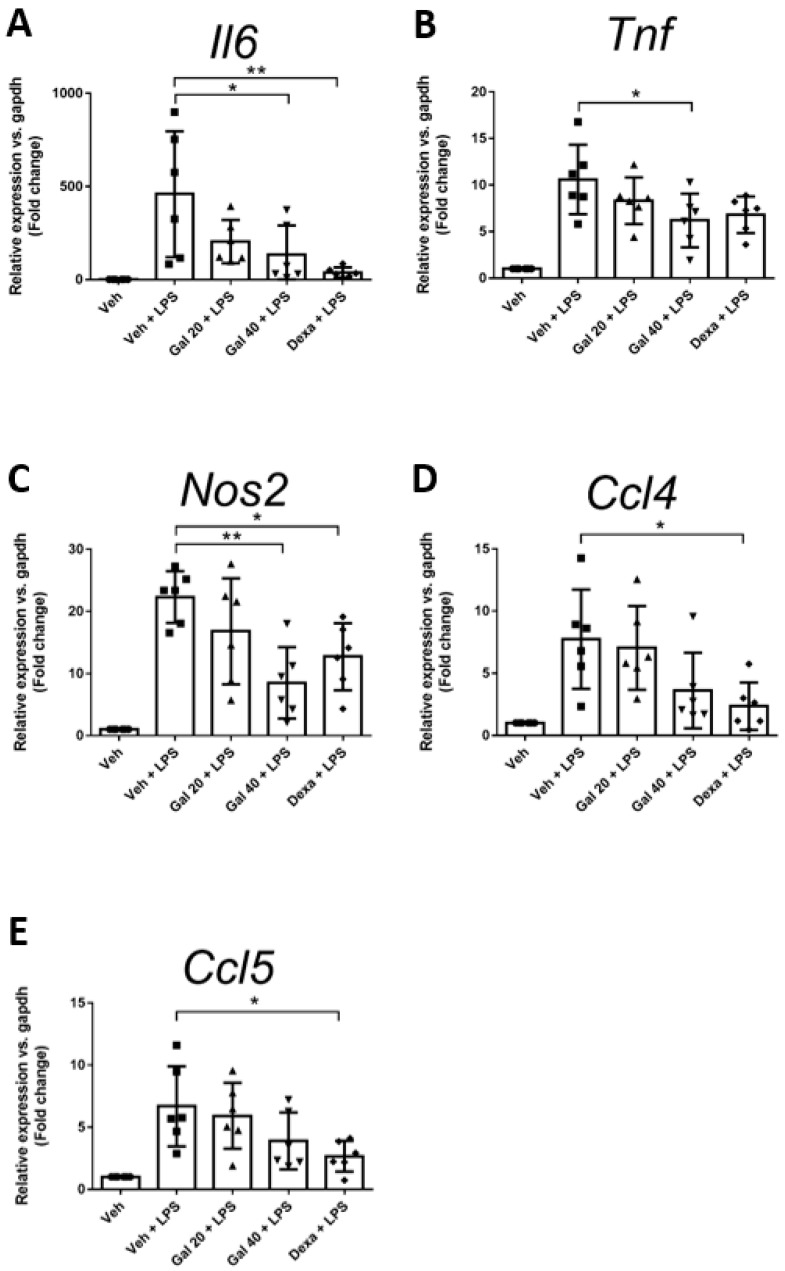
Galgravin downregulates the gene expression of IL-6, TNF-α, and iNOS in the lungs of LPS-challenged mice. The relative mRNA expression of IL-6 (**A**), TNF-α (**B**), iNOS (**C**), CCL4 (**D**), and CCL5 (**E**) in the lung tissues of treated mice collected at the end of experiments was measured using an RT-qPCR. The asterisk indicates significant differences from the LPS-treated vehicle control (*, *p* < 0.05; **, *p* < 0.01). CCL, C-C motif chemokine ligand.

To evaluate the suppressive effects of galgravin on inflammatory mediators in LPS-treated mice, the lung tissues of treated mice were collected and further analyzed for the gene expression of cytokines (IL-6, TNF-α), chemokines (CCL4, CCL5), and proinflammatory molecule (iNOS). Our results show that the intraperitoneal administration of galgravin (40 mg/kg) suppressed the gene expression of IL-6, TNF-α, and iNOS in the lung tissue of LPS-challenged mice (Figure 4A–C), whereas the expression of CCL4, and CCL5 in the lungs only showed a decreasing trend after galgravin treatment (Figure 4D,E).

### 2.5. Galgravin Attenuates Inflammatory Responses in Multiple Organs of LPS-Challenged Mice

**Figure 5:**
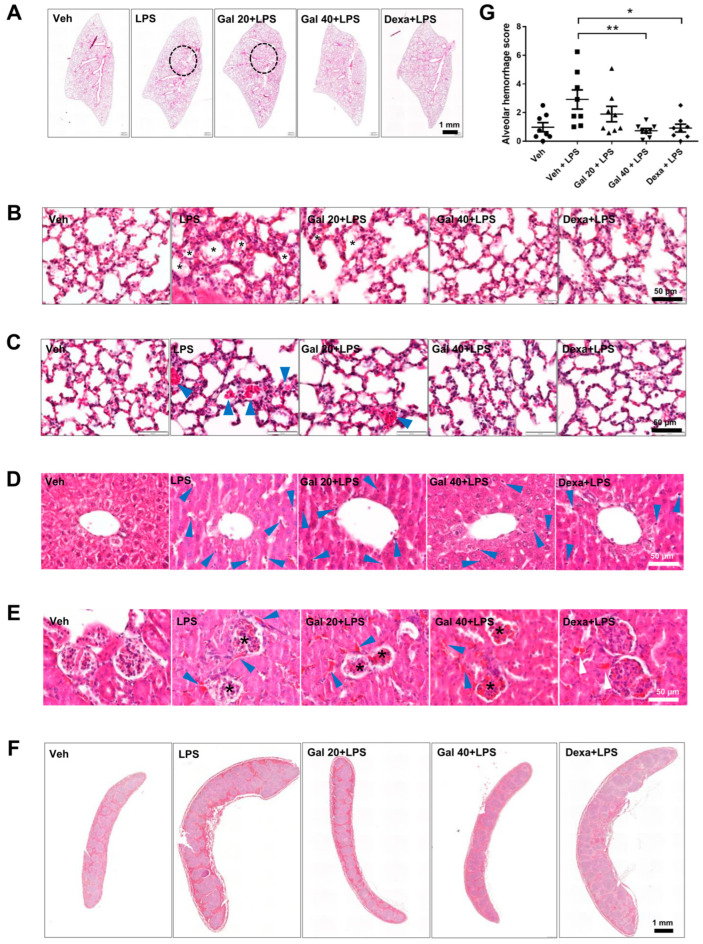
Intraperitoneal administration of galgravin improves pathological changes in LPS-challenged mice. (**A**) Representative images of H&E-stained lung tissues from vehicle-treated mice and LPS-treated mice without or with pretreatment with drugs are shown. The dashed circle denotes hilum congestion in the lung tissues. (**B**) The asterisk denotes alveolus with septal thickening in the lung tissues (original magnification, ×400). (**C**) The blue arrowhead denotes alveolar hemorrhage in the lung tissues (original magnification, ×400). (**D**) Representative images of liver sections stained with H&E for a histological assessment in various mouse groups. The blue arrowhead denotes inflammatory cells (original magnification, ×400). (**E**) Representative images of kidney sections stained with H&E for histological assessment in various mouse groups. The black asterisk denotes the reduction in Bowman’s space, and the blue arrowhead denotes a hemorrhage in the interstitial tissue (original magnification, ×400). (**F**) Representative images of spleen sections stained with H&E for histological assessment in various mouse groups. (**G**) The quantitation of the severity of alveolar hemorrhages in the lung tissues of vehicle-treated mice and LPS-treated mice without or with pretreatment with drugs. The alveolar hemorrhage was classified as group a: 6–10 red blood cells (RBCs), group b: 11–20 RBCs, or group c: > 20 RBCs in a single cut section of the alveolus. Twelve high-power field (HPF) images were analyzed in each mouse. The score was calculated as (a × 1 + b × 2 + c × 3)/12. The asterisk indicates significant differences from the LPS-treated vehicle control (*, *p* < 0.05; **, *p* < 0.01).

**Figure 6:**
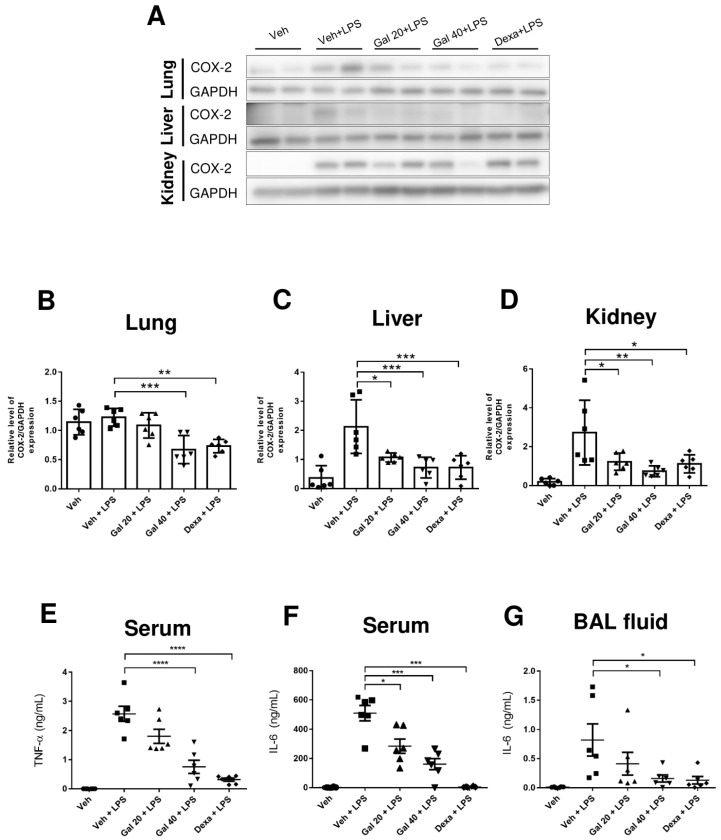
Galgravin reduces COX-2 expression in LPS-challenged mice. (**A**) The protein expression of COX-2 in various tissue lysates (50 μg) of vehicle-treated mice and LPS-treated mice without or with pretreatment with drugs was determined using Western blot analysis. (**B**–**D**) The quantification of COX-2 expression among different mouse groups. (**B**) Lung; (**C**) liver; and (**D**) kidney. (**E**) The quantitation on serum TNF-α levels of different groups of treated mice using ELISA. (**F**) The quantitation on serum IL-6 levels of different groups of treated mice using ELISA. (**G**) The quantitation on BAL fluid IL-6 levels of different groups of treated mice using ELISA. The asterisk indicates a significant difference versus the LPS-treated vehicle control (*, *p* < 0.05; **, *p* < 0.01; ***, *p* < 0.001; ****, *p* < 0.0001). BAL, bronchoalveolar lavage.

Because the lung, liver, and kidney are vital organs susceptible to LPS toxicity in mice [27], we further examined whether the intraperitoneal administration of galgravin attenuates LPS-induced histological changes in these organs of LPS-challenged mice. We found that galgravin improved hilum congestion, reduced alveolar septal thickening, and significantly diminished alveolar hemorrhage in the lung tissues of LPS-challenged mice (Figure 5A–C,G). In addition, inflammatory cells in the livers and hemorrhage in the kidney were also reduced by galgravin (Figure 5D,E). In addition, galgravin reduced LPS-induced splenomegaly (Figure 5F). Alternatively, we measured COX-2 levels in these tissues and observed that the intraperitoneal administration of galgravin significantly reduced the elevation of COX-2 in LPS-challenged mice (Figure 6A–D). Moreover, we further determined proinflammatory cytokines in the body fluids of LPS-challenged mice. Our results show that galgravin reduced the LPS-induced elevation of TNF-α and IL-6 in the serum (Figure 6E,F). Furthermore, the elevated IL-6 level in the bronchoalveolar lavage (BAL) fluid of LPS-challenged mice was also decreased by galgravin (Figure 6G). Finally, we conducted an alternative administration of galgravin at 80 mg/kg for five consecutive days via intragastric gavage, and results showed that this administration of galgravin also significantly decreased TNF-α in the serum (Supplementary Figure S1).

## 3. Discussion

In the present study, the anti-inflammatory effects of galgravin were investigated using LPS-activated RAW 264.7 macrophages, BMDM cells, and LPS-challenged mice. Briefly, elevated NF-κB and proinflammatory cytokines (TNF-α and IL-6) in LPS-activated RAW 264.7 macrophages, as well as the production of the iNOS and COX-2 protein, were downregulated by galgravin (Figure 1A,C,D and Figure 2). The elevated levels of TNF-α and IL-6 in LPS-activated BMDM cells were also reduced by galgravin (Figure 1E,F). In addition, galgravin prevented body weight loss in LPS-challenged mice (Figure 3B) and reduced proinflammatory cytokines (TNF-α and IL-6) (Figure 4A,B and Figure 6E–G; supplementary Figure S1G). To our knowledge, this is the first report to demonstrate the anti-inflammatory activity of galgravin in a sepsis animal model.

A significant anti-edematous effect of galgravin has been reported in rats receiving an oral gavage of galgravin at 10 or 20 mg/kg 30 min before a carrageenan injection [25]. Carrageenan, a seaweed polysaccharide widely used in the food industry, is known to induce paw edema in rodents’ hind limbs, serving as an in vivo model of local acute inflammation. While the molecular mechanism of such acute inflammation is not as straightforward as that of LPS, it has been suggested that carrageenan induces an inflammatory response by activating TLR2/6 and TLR4/6 pathways [28], which is different from the typical stimulation of LPS. Our observation of the galgravin-mediated suppression of proinflammatory cytokines and molecules in vitro and in vivo revealed the anti-inflammatory molecular mechanism of galgravin.

The low bioavailability (8.5%) of galgravin in rats receiving a single gavage of 20 mg/kg has been recently reported [17]. After the oral administration of galgravin, the plasma drug concentration peaked at 4 h and then decreased rapidly. Our observation that the oral administration of galgravin was less effective than intraperitoneal administration is likely due to insufficient oral bioavailability and a short half-life. Further optimization in the preparation, treatment protocol, and dosages of galgravin is necessary to improve oral drug efficacy in future animal studies.

Adult respiratory distress syndrome (ARDS) is a common pulmonary complication in septic shock, whereas LPS-induced acute lung injury in research animals is often used to mimic human ARDS. Notably, LPS is usually delivered via the intratracheal rather than the intraperitoneal route in most animal studies of sepsis-related ARDS. It has been shown that the tracheal instillation or inhalation of LPS is a direct pulmonary insult targeting the alveolar epithelium, and systemic LPS administration is an indirect insult targeting the pulmonary vascular endothelium [29]. In the mouse model of ARDS, the lung injury score is calculated by assessing neutrophils in the alveolar and interstitial space, proteinaceous debris filling the airspace, and alveolar thickening [30]. The present study did not detect significant differences in lung injury scores between the galgravin-treated and vehicle-treated LPS mice. However, alveolar hemorrhage, which results from red blood cell extravasation, showed a prominent improvement with galgravin treatment at 40 mg/kg. It is, therefore, suggested that galgravin may protect the pulmonary endothelium from LPS-induced injury.

The LPS-induced endotoxemia model is a well-accepted model for the in vivo inflammation study. However, there are several limitations in our LPS model. First, different LPS dosages (10, 20, 40 mg/kg, intraperitoneal) and treatment times (12, 16, 24 h) were tested for survival analysis. We chose an optimal LPS dosage of 20 mg/kg and 12 h, considering the balance between acceptable survival and illness severity. Due to the limitation of the animal number demand, we did not test a lower LPS dose of 10 mg/kg. Second, sepsis is a heterogeneous disease, and therefore, cecal ligation punctate (CLP) or colon ascendens stent peritonitis (CASP) might be more clinically relevant than LPS-induced endotoxemic mice to reproduce its heterogenicity.

Limitations: BMDMs were not characterized by staining for macrophage-specific markers due to the limited amount of BMDMs obtained. In addition, while no apparent changes in the number or morphology of BMDMs between the vehicle control and galgravin treatment groups were observed, we did not perform MTT assays on BMDMs after treatment with galgravin, and potential toxicity of galgravin in BMDMs was not evaluated.

## 4. Materials and Methods

### 4.1. Chemicals and Antibodies

The following reagents and chemicals were obtained from Sigma-Aldrich (St. Louis, MO, USA) as follows: LPS (*E. coli* O111:B4) for in vivo studies, the TRIzol reagent (T9424), and dexamethasone (D4902). The others were purchased from the following manufacturers: ultra-pure lipopolysaccharide (LPS) for in vitro studies (InvivoGen, San Diego, CA, USA); anti-COX-2 (Cell Signaling Technology, Danvers, MA, USA); anti-iNOS (Abcam, Cambridge, UK); anti-GAPDH (Millipore, Billerica, MA, USA); HRP-conjugated anti-rabbit IgG and HRP-conjugated anti-mouse IgG (Pierce, Rockford, IL, USA); PureLink^TM^ RNA Mini Kit (Invitrogen, Waltham, MA, USA); 10% neutral buffered formalin (Burnett, Taipei, Taiwan); and Mayer’s hematoxylin and Eosin alcohol solution (Muto Pure Chemicals, Tokyo, Japan).

### 4.2. Isolation of Galgravin from Piper kadsura

Galgravin was isolated and purified, as previously reported [31]. The stems of *Piper kadsura* were collected in December 2003 in Taiwan, and the voucher specimen number is 197,740. Briefly, the crushed stems of *P. kadsura* (8.5 kg) were extracted with methanol (60 L, three times) under reflux. The methanol extract was concentrated to dryness and partitioned between water and chloroform. The collected chloroform fraction was concentrated to dry powder (350 g) and then subjected to silica gel column chromatography (10 × 120 cm) with a gradient solvent constituted by ethyl acetate in *n*-hexane (0–100%) to obtain 14 fractions (No. 1–14). A solid precipitate was separated from fraction 7 and recrystallized from methanol to provide galgravin (3.5 g), which was then stored at −20 °C. The purity of galgravin was assessed using the HPLC method. Firstly, a galgravin solution in methanol (201 μg/mL) was prepared and subjected to liquid chromatography using a C18 reverse column (Cosmosil 5C_18_ AR-II, 4.6 × 250 mm) with a mobile phase constituted by water (A) and acetonitrile (B). The eluent gradient was set from 50% B to 90% B within 25 min, with an injected sample volume of 10 μL and a flow rate of 1 mL/min, and the chromatogram was recorded under UV 230 nm. The chromatogram (Supplementary Figure S2) indicates that the galgravin peak appeared at a retention time of 15.3 min, and its purity was 97%. For the in vitro study, galgravin was dissolved in a 100% DMSO solution, and a total of 1 μL of DMSO was used per 1 mL of the medium. For the in vivo-IP study, galgravin was dissolved in a 100% DMSO solution, and a total of 50 μL of DMSO was used. For the in vivo oral study, galgravin was dissolved in a 10% DMSO aqueous solution, and a total of 40 μL of DMSO was used.

### 4.3. Cell Culture of RAW 264.7 Macrophages

The RAW 264.7 murine macrophage cell line, purchased from the Bioresource Collection and Research Center (Hsinchu, Taiwan), was cultured in Dulbecco’s Modified Eagle Medium (DMEM) was supplemented with 10% of the heat-inactivated fetal bovine serum (FBS), 100 mg/mL of streptomycin, 100 units/mL of penicillin, 1 mM of sodium-pyruvate, and was 2 mM of L-glutamine. RAW 264.7/Luc-P1 is an LPS-responsive RAW 264.7 cell line containing an integrated reporter gene (pELAM1-Luc) established and cultured as described previously [26]. Briefly, the pELAM1-Luc consists of an NF-kB-responsive region from endothelial leukocyte adhesion molecule I (ELAM1), which is followed by firefly luciferase as the reporter gene. RAW264.7 cells were simultaneously co-transfected with pELAM1-Luc and pCI-puro plasmid, which expresses the puromycin-resistant gene. The transfected cells were grown in a puromycin-supplemented medium (6 μg/mL) to select puromycin-resistant single clones. After examining these clones using the luciferase assays for LPS responsiveness, the clone showing the strongest activity was designated RAW 264.7/Luc-P1. All cell culture reagents were purchased from Gibco (Grand Island, NY, USA).

### 4.4. Cell Culture of Bone Marrow-Derived Macrophages (BMDMs)

The bone marrow cells from the murine thigh bone were harvested and seeded at a density of 1 × 10^7^ cells in an uncoated 10 cm dish containing 20 ng/mL GM-CSF. A growth medium containing 20 ng/mL GM-CSF was added every other day for 6 days to induce differentiation. Afterward, BMDMs attached to the plates were collected and re-seeded for subsequent experiments.

### 4.5. Luciferase Reporter Assay

RAW 264.7/Luc-P1 cells (4 × 10^5^ cells/well in 24-well plates) were pretreated with a vehicle or galgravin for 1 h and then stimulated with 10 ng/mL LPS for 6 h. Afterward, the cells were incubated with 100 μL of the passive lysis buffer (Promega, Madison, WI, USA), and cell lysates (20 μL) were mixed with 100 μL of luciferin (Promega) immediately before luminescence detection using a Varioskan LUX spectral scanning multimode reader (Thermo Fisher, Waltham, MA, USA).

### 4.6. MTT Assay

RAW 264.7 cells (1 × 10^4^ cells/well in 96-well plates) were treated with a vehicle or galgravin (1, 3, 10, and 30 μM) for 24 h and then cultured in a growth medium containing the 3-(4,5)-dime- thylthiahiazo-(z-y1)-3,5-di-phenytetrazoliumromide (MTT) reagent (0.5 mg/mL) for 4 h and incubated with a solubilization buffer (12.5% sodium dodecyl sulfate, 45% dimethylformamide) for 16 h. The absorbance of the treated samples at A545 and A630 (as a reference) was then measured using a Sunrise ELISA reader (TECAN, Seestrasse, Switzerland).

### 4.7. Western Blot (WB)

The immunoblotting procedures were performed as described previously [32]. In brief, treated cells or liquid-nitrogen frozen tissues were lysed with an RIPA buffer (50 mM Tris (pH 7.4), 150 mM NaCl, 1% Nonidet P-40, 0.25% sodium deoxycholate, 5 mM EDTA (pH 8.0), and 1 mM EGTA) containing a protease inhibitor cocktail (Sigma-Aldrich). The protein lysates were quantitated using Bradford assays (Bio-Rad Laboratories, Hercules, CA, USA). An equivalent amount of the cell lysate (30 μg) or tissue lysate (50 μg) was analyzed using SDS-PAGE with the appropriate antibodies. Band images were further quantified using ImageJ version 1.53 (NIH, Bethesda, Rockville, MD, USA).

### 4.8. Measurement of Cytokines Using Enzyme-Linked Immunosorbent Assay (ELISA)

The supernatants of treated RAW 264.7 macrophages or BMDMs were collected for TNF-α (3 h post-LPS stimulation) and IL-6 measurement (8 h post-LPS stimulation). The serum from treated mice was collected for TNF-α (1 h post-LPS injection) and IL-6 measurements (12 h post-LPS injection). The BAL fluids from treated mice were collected for IL-6 analysis (12 h post-LPS injection). ELISA was performed using commercial ELISA kits following the manufacturer’s instruction (mouse TNF-α, DY410, R&D Systems, Minneapolis, MN, USA; mouse IL-6, 555240, BD, Franklin Lakes, NJ, USA).

### 4.9. Measurement of Gene Expression Using RT-PCR

Liquid-nitrogen frozen lung tissues (right cranial lobe) were homogenized, and total RNA was isolated using the TRIzol reagent (Sigma-Aldrich) and PureLink^TM^ RNA spin column kit (Invitrogen) according to the manufacturer’s instructions. The total RNA was reverse transcribed using HiScript III Q RT SuperMix (Vazyme Biotech, Nanjing, China). Then, the cDNA product was amplified via qPCR using a KAPA SYBR Fast qPCR Master Mix kit (Roche, Basel, Switzerland) and detected using a StepOne^TM^ Real-Time PCR System (Applied Biosystems, Waltham, MA, USA). The primers used are listed in Supplementary Table S1. All data were quantitated using 2^−ΔCt^ (ΔCt = Ct^Target gene^ − Ct^GAPDH^; Ct: cycle number when the fluorescent value of the sample was equal to the threshold value).

### 4.10. Animal Model

C57BL/6 male mice were purchased from the National Laboratory Animal Center (Taipei, Taiwan) and maintained at the animal facility of the National Yang Ming Chiao Tung University. An adequate temperature, humidity, and a light-dark 12/12 h circle were maintained. All mice had free access to drinking water and their diet. The endotoxemia mouse model was used to induce inflammatory responses as described previously [33]. Briefly, an injection with purified LPS in mice leads to the systemic activation of innate immunity. With increasing doses of LPS, the mice display physiological and biochemical changes that are similar to the outcome caused by the severe infection of Gram-negative bacteria in humans. Typically, the increased circulating levels of TNF-α and IL-6 are features of sepsis in humans and the LPS-induced endotoxemia mouse model. Prior to the galgravin treatment experiment, the survival curve analysis of mice was performed for various LPS dosages, and LPS 20 mg/kg was also used (Supplementary Figure S3). In experiments with the intraperitoneal administration of galgravin, male mice at the age of 6–7 weeks old were divided (*n* = 6 for each group) into the vehicle (DMSO + saline), LPS (DMSO + LPS 20 mg/kg), galgravin 20 + LPS (galgravin 20 mg/kg + LPS 20 mg/kg), galgravin 40 + LPS (galgravin 40 mg/kg + LPS 20 mg/kg), and dexamethasone + LPS groups (dexamethasone 10 mg/kg + LPS 20 mg/kg). Dexamethasone was used as a positive control [34]. Thirty minutes after vehicle or drug administration, saline or LPS was delivered to the mice intraperitoneally. Blood was drawn at 1 h post-LPS injection for TNF-α measurement. After 12 h post-LPS injection, the rectal temperature and body weight were recorded, followed by the sacrifice of mice. Blood was collected for the IL-6 measurement and serum biochemistry analysis, including the ALT and creatinine measurement, which was carried out at the end of the experiments. The lungs, livers, kidneys, and spleens were dissected for subsequent analysis. All investigations related to animal studies were approved by the Institutional Animal Care and Use Committee (IACUC NO. 1100406, 2021) at National Yang Ming Chiao Tung University (Taipei, Taiwan).

### 4.11. Bronchoalveolar Lavage (BAL) Fluid Analysis

Under microscopic visualization using a Zeiss Stemi SV 6 dissection microscope, a 24 G IV cannula (Introcan Certo) was indwelled to the lower part of the trachea. Then, 0.8 mL cold PBS was infused via trachea with the lungs in situ. The BAL fluid was withdrawn with syringe aspiration twice. After centrifugation at 2000 rpm for 5 min, the supernatants were collected for cytokine measurement.

### 4.12. Histological Analysis

Tissues from the sacrificed mice were fixed with 10% buffered formalin before being dehydrated stepwise in a graded series of alcohol solutions, ending with pure 100% alcohol and then xylene. Formalin Fixed Paraffin-Embedded (FFPE) samples were obtained using a standard procedure. The sliced sections, 4 μm in thickness, were stained with hematoxylin and eosin (H&E). Olympus BX 63 optical microscope (Olympus Optical Ltd., Tokyo, Japan) was used to capture the images.

### 4.13. Statistical Analysis

In vitro data are the mean ± SEM (*n* = 3). In vivo data are presented as the mean ± SEM (*n* = 6). One-way ANOVA followed by post hoc Turkey’s test was used to determine the significance of between-group differences. Statistical significance was set at a *p*-value of < 0.05.

## 5. Conclusions

Our present study provides in vitro and in vivo evidence that galgravin can act as an anti-inflammatory agent against LPS-induced inflammation. It is, therefore, suggested that galgravin, as a natural product isolated from *P. kadsura*, may warrant further investigation as a pharmaceutical agent for treating sepsis-related inflammatory diseases.

## Data Availability

The data are contained within the article.

## Supplementary Materials

The following supporting information can be downloaded at: https://www.mdpi.com/article/10.3390/ijms242316572/s1.
