# Supplementary material for: Galgravin Isolated from Piper kadsura Ameliorates Lipopolysaccharide (LPS)-Induced Endotoxemia in Mice
Source: Int J Mol Sci. 2023 Nov 21;24(23):16572. doi: 10.3390/ijms242316572 (PMC10706620; doi:10.3390/ijms242316572)
Supplement: Supplementary file 1 [file ijms-24-16572-s001.zip › Supplementary Figures.pdf]

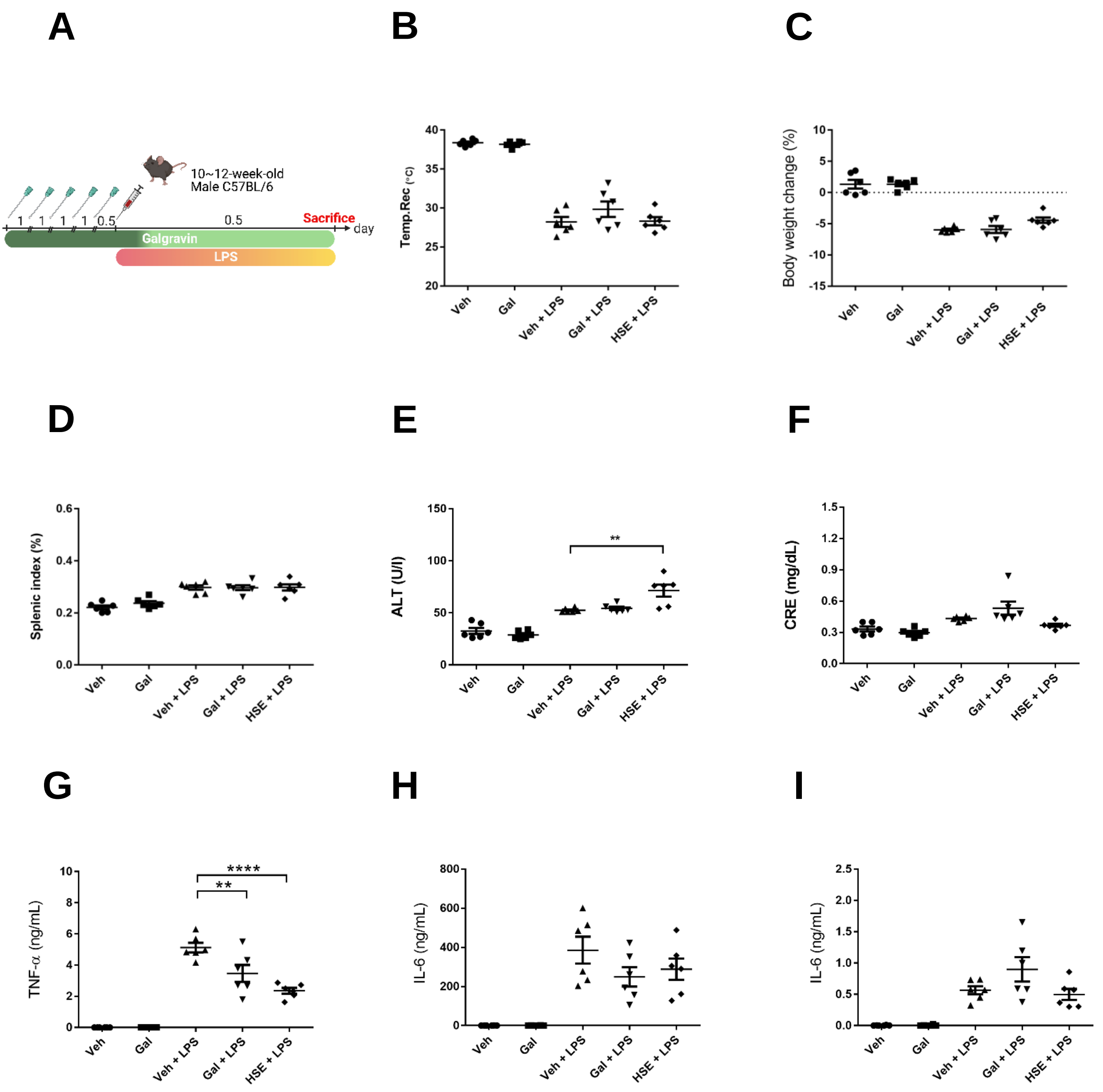


**Supplementary Figure S1. Oral administration of galgravin suppressed serum TNF- αin LPS-treated mice.** (A) The experimental scheme of LPS-induced endotoxemia mouse model with oral administrations of galgravin for five consecutive days before LPS treatment. N=6 for each group. *Hypericum sampsonii* extract (HSE) was used as a positive control. (B) Rectal temperatures of the vehicle-treated mice and the LPS-treated mice without or with pretreatment of drugs were recorded at sacrifice. (C) Body weight changes of varying mouse groups were recorded before sacrifice. (D) The splenic index (%) of various mouse groups was calculated from the percentage of splenic weight to body weight at sacrifice. (E) Serum ALT of various mouse groups was measured to evaluate their degree of hepatotoxicity. (F) Serum CRE of various mouse groups was measured to assess their nephrotoxicity.(G) Serum TNF- αof various mouse groups at 1 h post-LPS. (H) Serum IL-6 of various mouse groups at sacrifice. (I) BAL fluid IL-6 of various mouse groups at sacrifice. LPS dosage was 20 mg/kg. GAL dosage was 80 mg/kg. HSE dosage was 200 mg/kg. One-way ANOVA followed by post hoc Turkey's test was used to determine the significant differences between mouse groups. The asterisk indicates significant differences from the LPS-treated vehicle control (**, $p < 0.01$; ****, $p < 0.0001$).

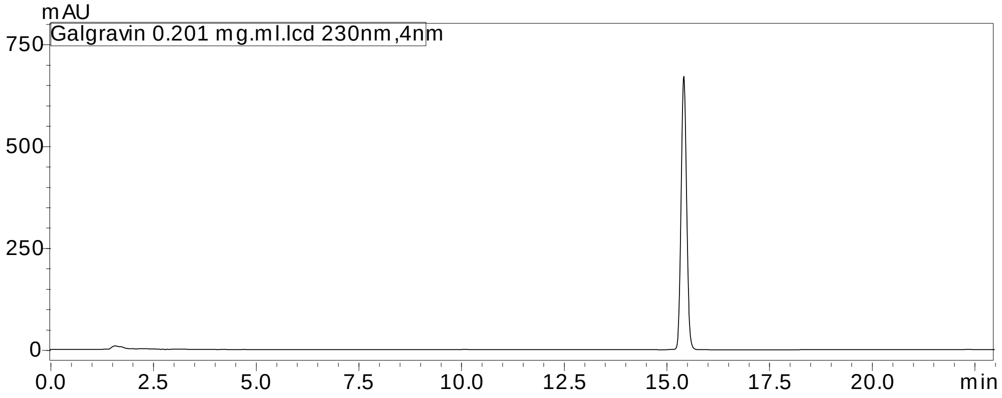


**Supplementary Figure S2. HPLC chromatogram of galgravin under UV 230 nm detection.**

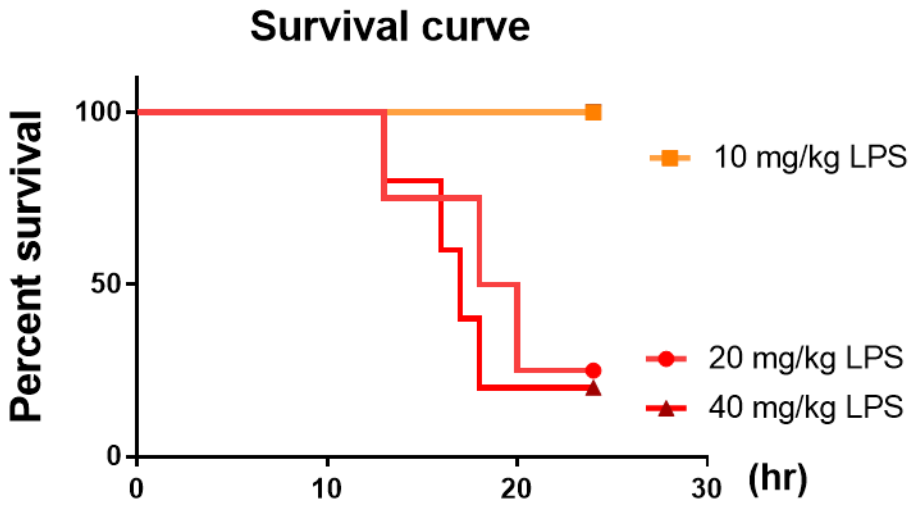


**Supplementary Figure S3. Effects of varied LPS doses on the mortality of LPS-challenged mice.** Male C57BL/6 mice were injected intraperitoneally with varied doses of LPS (10, 20, 40 mg/kg) and the survival rate was recorded at different time intervals.
