# Supplementary material for: Galgravin Isolated from Piper kadsura Ameliorates Lipopolysaccharide (LPS)-Induced Endotoxemia in Mice
Source: Int J Mol Sci. 2023 Nov 21;24(23):16572. doi: 10.3390/ijms242316572 (PMC10706620; doi:10.3390/ijms242316572)
Supplement: Supplementary file 1 [file ijms-24-16572-s001.zip › Supplementary Table S1.pdf]

Supplementary Table 1. The primers used in real-time PCR

Primer	Sequence
mIL-6-F’	TAGTCCTTCCTACCCCAATTTCC
mIL-6-R’	TTGGTCCTTAGCCACTCCTTC
mTNF-F’	CAGGCGGTGCCTATGTCTC
mTNF-R’	CGATCACCCCGAAGTTCAGTAG
mNOS2-F’	GTTCTCAGCCCAACAATACAAGA
mNOS2-R’	GTGGACGGGTCGATGTCAC
mCCL4-F’	TTCCTGCTGTTTCTCTTACACCT
mCCL4-R’	CTGTCTGCCTCTTTTGGTCAG
mCCL5-F’	GCTGCTTTGCCTACCTCTCC
mCCL5-R’	TCGAGTGACAAACACGACTGC
mGAPDH-F’	TGTGATGGGTGTGAACCACGAG
mGAPDH-R’	TGCTGTTGAAGTCGCAGGAGAC
